# Genetic diversity, population structure and anthracnose resistance response in a novel sweet sorghum diversity panel

**DOI:** 10.3389/fpls.2023.1249555

**Published:** 2023-10-20

**Authors:** Hugo E. Cuevas, Joseph E. Knoll, Louis K. Prom, Lauren R. Stutts, Wilfred Vermerris

**Affiliations:** ^1^ USDA-ARS, Tropical Agriculture Research Station, Mayagüez, Puerto Rico; ^2^ USDA-ARS, Crop Genetics and Breeding Research, Tifton, GA, United States; ^3^ USDA-ARS, Southern Plains Agriculture Research Center, College Station, TX, United States; ^4^ Graduate Program in Plant Molecular & Cellular Biology, University of Florida, Gainesville, FL, United States; ^5^ Department of Microbiology & Cell Science and UF Genetics Institute, University of Florida, Gainesville, FL, United States

**Keywords:** anthracnose, genetic diversity, GWAS, population structure, *Sorghum bicolor*, *Colletotrichum sublineolum*

## Abstract

Sweet sorghum is an attractive feedstock for the production of renewable chemicals and fuels due to the readily available fermentable sugars that can be extracted from the juice, and the additional stream of fermentable sugars that can be obtained from the cell wall polysaccharides in the bagasse. An important selection criterion for new sweet sorghum germplasm is resistance to anthracnose, a disease caused by the fungal pathogen *Colletotrichum sublineolum.* The identification of novel anthracnose-resistance sources present in sweet sorghum germplasm offers a fast track towards the development of new resistant sweet sorghum germplasm. We established a sweet sorghum diversity panel (SWDP) of 272 accessions from the USDA-ARS National Plant Germplasm (NPGS) collection that includes landraces from 22 countries and advanced breeding material, and that represents ~15% of the NPGS sweet sorghum collection. Genomic characterization of the SWDP identified 171,954 single nucleotide polymorphisms (SNPs) with an average of one SNP per 4,071 kb. Population structure analysis revealed that the SWDP could be stratified into four populations and one admixed group, and that this population structure could be aligned to sorghum’s racial classification. Results from a two-year replicated trial of the SWDP for anthracnose resistance response in Texas, Georgia, Florida, and Puerto Rico showed 27 accessions to be resistant across locations, while 145 accessions showed variable resistance response against local pathotypes. A genome-wide association study identified 16 novel genomic regions associated with anthracnose resistance. Four resistance loci on chromosomes 3, 6, 8 and 9 were identified against pathotypes from Puerto Rico, and two resistance loci on chromosomes 3 and 8 against pathotypes from Texas. In Georgia and Florida, three resistance loci were detected on chromosomes 4, 5, 6 and four on chromosomes 4, 5 (two loci) and 7, respectively. One resistance locus on chromosome 2 was effective against pathotypes from Texas and Puerto Rico and a genomic region of 41.6 kb at the tip of chromosome 8 was associated with resistance response observed in Georgia, Texas, and Puerto Rico. This publicly available SWDP and the extensive evaluation of anthracnose resistance represent a valuable genomic resource for the improvement of sorghum.

## Introduction

Sorghum [*Sorghum bicolor* (L.) Moench] is the fifth most widely cultivated cereal worldwide behind wheat (*Triticum aestivum* L.), maize (*Zea mays* L.), rice (*Oryza sativa* L.) and barley (*Hordeum vulgare* L.) (FAOSTAT, 2022). Sorghum originated in the northeast of Africa, but five independent re-domestication processes on this continent led to five botanical races (Bicolor, Caudatum, Durra, Guinea and Kafir) (Smith and Frederiksen, 2000), defined by their inflorescence architecture and environmental adaptation. These re-domestication events led to a highly genetically diverse crop (grain, forage, high-biomass, and sweet sorghum) with a broad range of end uses. In addition to its versatility, sorghum is well positioned for sustainable agricultural production due to its drought tolerance, adaptability to widely different climatic conditions, and its ability to grow on low-fertility soils.

Sweet sorghum is characterized by tall, juicy stalks that contain a high concentration of soluble sugars. Since the 1970s sweet sorghum has been considered an attractive feedstock for biofuel production (Jackson et al., 1980), initially to reduce dependence of foreign oil, and more recently to reduce the emission of greenhouse gases, whereby fermentable sugars can be obtained from both the juice and the cell wall polysaccharides in the bagasse (Rooney et al., 2007; Umakanth et al., 2012; van Rijn et al., 2018). Even though multiple research projects have focused on improving the production of ethanol from sweet sorghum (Antonopoulou et al., 2008; Wu et al., 2010; Yu et al., 2012), further improvements in ethanol yield on a per-area basis are possible by exploring and exploiting the genetic diversity of sweet sorghum (Murray et al., 2008a; Murray et al., 2008b; Murray et al., 2009; Wang et al., 2009; Cuevas et al., 2015).

The United States Department of Agriculture, Agriculture Research Service (USDA-ARS) and its National Plant Germplasm System (NPGS) maintains a sorghum germplasm collection that includes >41,860 accessions from 114 countries. Approximately 2,180 of these accessions have been classified as sweet sorghum based on their name, phenotypic data, or breeding program source (Gary Pederson, *personal communication*). This sweet sorghum subset also contains most of the sweet sorghum germplasm developed and released in the U.S. until the middle of the twentieth century (Wang et al., 2009). Genetic and population structure analysis using a limited number of accessions and genetic markers revealed a narrow genetic diversity among improved sweet sorghum accessions (Ali et al., 2008; Murray et al., 2009; Wang et al., 2009). In fact, most of the sweet sorghum germplasm developed in the U.S. traces its origins to just six African landraces (Murray et al., 2009) that could be the original donors of valuable alleles for traits relevant for biofuel production (Cuevas et al., 2015). However, it is necessary to identify the most valuable germplasm for breeding programs based on both phenotypic and genetic analyses.

The economic impact of sorghum foliar disease is larger in biomass energy feedstocks than in grain types. Anthracnose [*Colletotrichum sublineolum* P. Henn., in Kabat and Bubák (syn. *C. graminicola* (Ces.) G. W. Wilson] is among the most damaging fungal diseases because it affects all the aerial tissues of the plant (i.e. leaf, stem and inflorescence) (Thakur and Mathur, 2000). The disease is most prevalent in warm and humid production regions where biomass sorghum is more likely to be grown (Gautam et al., 2020). The preferred strategy to control anthracnose is through incorporation of resistance genes (Stutts and Vermerris, 2020). Today, multiple sources of resistance have been identified in NPGS tropical germplasm (Erpelding, 2011; Prom et al., 2011; Cuevas et al., 2014; Cuevas et al., 2019; Cuevas and Prom, 2020) and temperate adapted grain lines (Prom et al., 2016; Cuevas et al., 2018), but less is known about sources of resistance in sweet sorghum germplasm (Cuevas et al., 2015). The identification of novel anthracnose resistance sources and genetic loci present in sweet sorghum germplasm will expedite the development of new anthracnose-resistant sweet sorghum cultivars and hybrids by avoiding the time-consuming introgression breeding approaches that use non-sweet sorghums as donors of resistance alleles. Therefore, the identification of new resistant germplasm is necessary to pyramid multiple resistance genes with the objective of eliciting an effective resistance response across environments or in environments where multiple pathotypes exist.

The NPGS sweet sorghum collection is the primary source of genetic diversity for the development of sweet sorghum varieties for biofuel production. The establishment of a representative subset of this collection could encourage its utilization in breeding programs and help conserve it. In the current study, we genetically characterized 272 NPGS sweet sorghum accessions via GBS to: 1) determine the population structure and genetic relationship among accessions; 2) identify new sources of anthracnose resistance across four geographic locations; and 3) identify resistance loci using GWAS to understand host/pathogen interactions. This new, publicly available germplasm collection and its genomic characterization provide a novel resource that will be complementary to the sorghum association panel (SAP; Casa et al., 2008) and the bioenergy association panel (BAP; Brenton et al., 2016) for developing new sorghum cultivars.

## Materials and methods

### Germplasm

A core set of 272 accessions from the NPGS sweet sorghum germplasm collection that included 99 improved germplasm accessions, 132 landraces from 22 countries, and 41 accessions from unknown origin were selected based on sugar concentration [≥9.0°Brix; Germplasm Resources Information Network (accessed August 23,2023)], sorghum race and place of origin (**Supplementary Table S1**). This core set [named after herein as Sweet Sorghum Diversity Panel (SWDP)] included the five major sorghum races (Bicolor, Caudatum, Kafir, Durra and Guinea), represented ~10% of the NPGS sweet sorghum germplasm collection, and only shared 15 and 5 accessions with the BAP (Brenton et al., 2016) and SAP (Casa et al., 2008), respectively.

### Field experiments

The SWDP and five reference lines that included both anthracnose resistant (SC112-14 and SC784-5) and susceptible accessions (RTx430, BTx623, and Early Honey) were planted in April 2017 and December 2018 at the research farm of the USDA-ARS Tropical Agriculture Research Station in Isabela, Puerto Rico, U.S.A., for evaluation and seed production. In summer 2017 and 2018, the SWDP and reference lines were planted at the Black Shank Farm of the University of Georgia in Tifton, Georgia, U.S.A. (31°29’58.9”N, 83°32’53.8”W), at the Research Farm of Texas A&M University at College Station, Texas, U.S.A. (30°31’55.5”N, 96°25’23.7”W), and at the University of Florida North Florida Research and Education Center-Suwannee Valley near Live Oak, Florida, U.S.A. (30°18’47.8”N, 82°54’07.8”W). The accessions at the four locations were planted in a randomized complete block design with two blocks in single-row plots of 3.1 m in length with 20-30 plants and 0.9 m between rows.

### Anthracnose resistance

Even though the high relative humidity at these four locations makes anthracnose an endemic sorghum disease, several plants per plot were manually inoculated to have a uniform disease distribution in the field. The inoculations were similar to those described by Prom et al. (2009), where fungal cultures were prepared with different isolates of *C. sublineolum* that represent the pathotypes present at each location. These isolates were used to colonize autoclaved sorghum seeds that were later placed into the leaf whorl of 30-45 day- old plants. The anthracnose resistance response of each plot was determined approximately 30-45 days after flowering (hard-dough state to physiological maturity). The plots were visually inspected and the most infected plant was used to determine the diseases rating using the 1-to-5 scale as follows: 1 = no symptoms or chlorotic flecks on leaves; 2 = hypersensitive reaction on inoculated leaves, but no acervuli in the center; 3 = infected bottom leaves with acervuli formation in at least one plant within the plot; 4 = necrotic lesions with acervuli on bottom leaves showing spreading to middle leaves in at least one plant within the plot; and 5 = most leaves dead because of infections and flag leaf infected in at least one plant within the plot (Prom et al., 2009). An analysis of variance (ANOVA) for anthracnose resistance response was performed using the *proc mixed covtest* method *type3* procedure of SAS (version 9.4, SAS Institute, Cary, NC). In the mixed linear model for such ANOVA the locations and years were treated as fixed effects and accessions as a random effect with the following model: *A* = μ + *Y* + *L* + *L×Y* + *G* + *G×L* + *ϵ*; where *A* is anthracnose resistance response, μ is the common effect, *Y* represents the effect of the year, *L* is the location effect, *Y×L* is location *×* year interaction, *G* is the effect of the accessions, *L×G* is the location *×* accession interaction and *ϵ* is the error term (residual).

The anthracnose resistance response of each accession was also categorized as resistant (score ≤ 2.0) or susceptible (score > 2.0), and the frequency of resistant accessions was determined for each population and the SWDP as a whole in each location. To examine if any of the populations within the SWDP had been selected for anthracnose resistance, we performed a χ^2^ test using the observed number of resistant genotypes within each population and the expected number based on the frequencies in the entire SWDP. Segregation distortion against the expected ratio [χ^2^ *P*(value) < 0.01] was considered a signature of selection. To determine the correlation between photoperiod-sensitivity and anthracnose resistance, the flowering time of each plot at Tifton, GA was recorded as the number of days post planting until 50% of the plants within a plot reached anthesis. The Pearson correlation coefficient was calculated in SAS (Cary, NC) using an assigned value of 300 days to photoperiod-sensitive accessions (i.e. plants that did not flower).

### DNA isolation and preparation of a genotyping-by-sequencing library

Genomic DNA was isolated from seedlings of the SWDP using the method of Guillemaut and Marechal-Drouard (1992) and purified using the ZR96 DNA Clean & Concentrator-5 kit (Zymo Research, Irvine, CA, USA). A genotyping-by-sequencing (GBS) library was prepared using the restriction enzyme *Ape*K1 for digestion (Elshire et al., 2011) and sequenced in three lanes of an Illumina HiSeq 2500 platform at the Biotechnology Center of the University of Wisconsin, Madison, WI, USA.

### SNP calling and genomic characterization

The Tassel 5.0 v2 GBS pipeline (Glaubitz et al., 2014) was used to call SNPs using the most recent version of the BTx623 sorghum genome [version v.3; www.sorghumbase.com; (Paterson et al., 2009)] (**Supplementary Figure S1**). Raw genotypes with minor allele frequencies (MAF) > 0.01 and missing data < 0.25 were filtered to retain 171,954 SNPs that were imputed using Beagle 4.1 (Browning and Browning, 2016) with a probability call of >0.80. The number of SNPs within genes, in proximity of genes (i.e. within 5 kb upstream or downstream of a gene), and in intergenic regions, as well as the number of synonymous and non-synonymous SNPs were determined using the Variant Effect Predictor (McLaren et al., 2016), as implemented in EnsemblPlants (http://plants.ensembl.org/).

### Population structure

Population structure of the SWDP was assessed using the model-based clustering method implemented in STRUCTURE 2.1 (Pritchard et al., 2000). The genotypic data were thinned to 2,345 unlinked SNPs (r^2^ <0.10) using PLINK (Purcell et al., 2007) and used in the analysis. For each *k* value set from 1 to 15, three independent analyses were run using an admixture model with correlated frequencies, 25,000 burn-in periods, and 125,000 Monte Carlo Markov Chain (MCMC). The actual number of populations was determined using the *ad hoc* statistic Δ*k* based on the rate of change in the log probability of data between successive *k* values (Evano et al., 2005), as implemented by the Structure Harvester software (Earl and Vonholdt, 2012). The ancestry membership coefficient of each accession was obtained by matching the three independent runs of the selected *k* values in CLUMPP (Jakobsson and Rosenberg, 2007). Accessions with an ancestry membership coefficient >0.60 were assigned to their corresponding population. Principal component analysis (PCA) was conducted using Tassel 5.0.

### Cluster analysis

The identical-by-state (IBS) genetic distances among SWDP accessions were calculated in Tassel 5.0 using the 2,345 unlinked SNPs. The resulting matrix was subjected to a clustering analysis using neighbor-joining and visualized using Interactive Tree of Life (Letunic and Bork, 2011). The pairwise fixation indexes (F_ST_) among populations were estimated in the R package HIERFSTAT (Goudet, 2005).

The genetic relationship between SWDP and the BAP was investigated through clustering and PCA analysis. Genotyping information of the BAP was obtained from the Dryad Data Repository (Hu et al., 2019) and merged with SWDP resulting in a total of 94,999 common SNPs. These genotyping data were thinned to 7,792 unlinked SNPs (r^2^ <0.10) using PLINK (Purcell et al., 2007) and used in the analysis. The IBS pairwise genetic distance and PCA analysis among the 609 accessions from SWDP and BAP were calculated in Tassel 5.0 (Glaubitz et al., 2014). The resulting matrix was subjected to a clustering analysis using neighbor-joining and visualized using Interactive Tree of Life (Letunic and Bork, 2011).

### Genome-wide association analysis

Genome-wide association analyses were performed using 132,399 SNPs with MAF ≥ 0.05 and the Bayesian-information and Linkage-disequilibrium Iteratively Nested Keyway (BLINK) model implemented in the R-package GAPIT (Lipka et al., 2012). Log quantile-quantile (Q-Q) *p-*value plots were examined to determine that the inclusion of the first three ancestry coefficients adequately controlled for the population structure and family relatedness. Association analyses were performed for photoperiod-sensitivity (i.e. non-flowering under short days) and anthracnose resistance response for each location. The empirical significance threshold for BLINK based on the false discovery rate (Benjamini and Hochberg, 1995) as implemented in GAPIT using a cutoff p-value ≤0.01. Manhattan and Q-Q plots were visualized using the R package qqman (Turner, 2014).

The genomic regions in linkage disequilibrium with associated SNPs were delimited using the *block* function of PLINK (Purcell et al., 2007) and plots were visualized using the R package LD heatmap (Shin et al., 2006). The candidate genes within these genomic regions were identified based on the annotation of the sorghum reference genome BTx623 v.3.1.1 (https://phytozome-next.jgi.doe.gov/). Functional annotations included protein domains encoded by putative genes and associated PlantFAMs identified via hmmsearch (Finn et al., 2011) that evaluate remote protein homology as implemented in Phytozome 13.

## Results

### Anthracnose resistance in the SWDP

Analysis of the anthracnose resistance response of the SWDP across the four locations revealed that the resistance response of multiple accessions is determined by *C. sublineolum* pathotypes (**Table 1**; **Supplementary Table S1**). The ANOVA indicated year, location, accessions, and the interactions year × location and accessions × location have statistically highly significant effects (P ≤0.001; **Supplementary Table S2**) on the anthracnose resistance response. We observed that the frequency of resistant accessions in Puerto Rico (130 accessions) was higher than in Texas (90 accessions), Florida (80 accessions) and Georgia (67 accessions). Twenty-six accessions were consistently resistant (≤2.0; no acervuli observed) across locations, and this group of accessions includes both landraces and advanced breeding germplasm.

**Table 1:** Anthracnose incidence of 224 accessions from the NPGS sweet sorghum diversity panel (SWDP) evaluated in 2018 and 2019 at Florida (FL), Georgia (GA), Texas (TX), and Puerto Rico (PR). The accessions were divided according to the population structure analysis.

	SWDP		Population 1			Population 2			Population 3			Population 4			Admixed		
Location	R^1^	S^1^	R^1^	S^1^	*P-value^2^*	R^1^	S^1^	*P-value^2^*	R^1^	S^1^	*P-value^2^*	R^1^	S^1^	*P-value^2^*	R^1^	S^1^	*P-value^2^*
FL	80	144	17	10	0.00	9	64	0.00	23	12	0.00	5	13	0.48	26	45	0.87
GA	67	157	16	11	0.00	7	66	0.00	19	16	0.00	5	13	0.84	20	51	0.75
TX	90	134	12	15	0.65	38	35	0.04	13	22	0.71	6	12	0.55	21	50	0.07
PR	130	93	17	10	0.62	49	24	0.13	18	16	0.53	9	9	0.47	37	34	0.29
Overall	27	196	7	20	0.02	4	69	0.07	5	30	0.75	3	15	0.60	8	63	0.75

^1^R and S refers to resistant and susceptible to anthracnose.

^2^P-value of the chi-squared test

For each location, a chi-squared test (*χ^2^*) was used to determine if the frequency of resistant accessions in each population deviated from the frequency of resistant accessions observed among the SWDP in each location.

### Genomic diversity

The GBS analysis of the SWDP identified 171,954 SNPs with an average of one SNP per 4,071 kb (**Table 2**). These SNPs tagged 24,892 annotated genes (within 5 kb upstream or downstream) and included 22,975 non-synonymous mutations, suggesting the presence of novel alleles. After removing SNPs with a frequency lower than 0.05 (i.e. rare alleles), 132,399 SNPs were retained with an average LD block of 11,020 kb across the genome. Moreover, we observed that 16,178 SNPs were contained within 490 LD blocks ranging from 50-200 kb. The genomic comparison of this panel against the BAP identified 94,999 common SNPs. Therefore, the SWDP could be used to expand the genetic diversity of the BAP and to increase the statistical power to detect SNPs associated with under-represented traits of interest.

**Table 2:** Genomic distribution of 171,954 SNPs identified among the NPGS sweet sorghum diversity panel.

	Chromosome										
	1	2	3	4	5	6	7	8	9	10	Total
Overlapped genes	4099	3235	3330	2759	1833	2204	1725	1530	2018	2159	24892
5_prime_UTR_variant	1916	1340	1518	1271	852	1041	849	786	900	982	11455
3_prime_UTR_variant	1240	1096	1128	1010	644	819	562	534	668	738	8439
Missense variant	3221	3139	2813	2195	2478	1998	1588	1855	1769	1919	22975
Synonymous variant	3164	2911	2753	2261	2025	1927	1513	1694	1758	1721	21727
Intron variant	3098	2484	2668	2088	1324	1916	1310	1298	1566	1560	19312
Upstream gene variant	5034	4259	4191	3879	2548	3083	2189	2112	2766	2687	32748
Downstream gene variant	2224	2001	1720	1518	1640	1202	1186	1092	1190	1356	15129
Intergenic variant	3334	4474	3533	3515	4838	3720	3302	4122	3170	3465	37473
Others	398	362	366	286	194	229	202	185	246	228	2696

### Population structure

**Figure 1:**
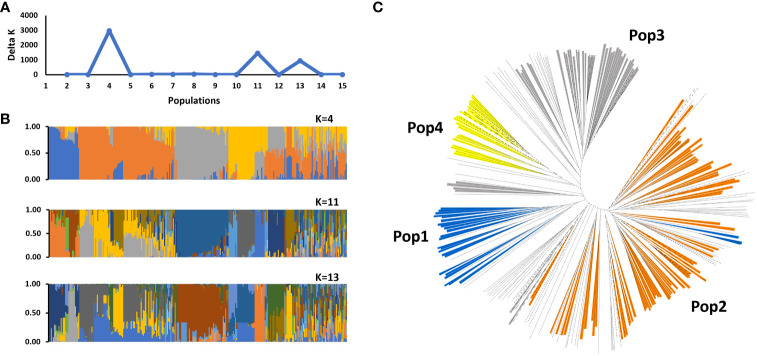
Population structure analysis of the NPGS sweet sorghum diversity panel (SWDP) using 2,345 unlinked genome-wide SNPs. **(A)** Estimation of the number of populations in the SWDP based on the analysis in STRUCTURE, with Δ*k* values ranging from 1 to 16. **(B)** Hierarchical organization of genetic relatedness of SWDP for K values of 4, 11 and 13. **(C)** Unrooted neighbor-joining tree of SWDP.

**Figure 2:**
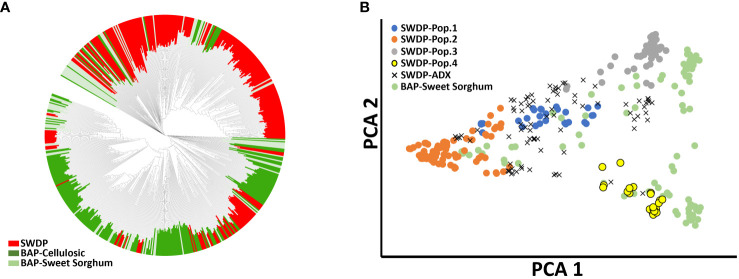
Phylogenetic analysis of 337 accessions from the sorghum bioenergy panel (BAP) and 272 accessions from NPGS sweet sorghum diversity panel (SWDP). **(A)** Unrooted neighbor-joining tree based on the accessions from SWDP and BAP. **(B)** Principal component analysis of the SWDP and sweet sorghum germplasm present in the BAP.

Population structure analysis revealed that the SWDP could be stratified into four populations and one admixed group (**Figure 1**). In total, 188 accessions were assigned to one of these four populations, while 84 accessions were an admixture. This population structure was consistent in the neighbor-joining tree analysis where the four populations were dispersed into different clades separated by admixed clades. We observed that the population structure could be associated with the sorghum domestication process. Racial classifications based on panicle architecture showed that most of the accessions in population 1 belong to the Guinea race. In population 2, Caudataum, Guinea and their intermediates (Caudatum/Guinea) constituted 77% of the accessions. Most of the accessions in population 3 (81%) belonged to Caudatum, Durra and their intermediate (Caudatum/Durra). The Ethiopian accessions classified into the Durra race were grouped within population 4. The F_ST_ values showed that population 2 is highly genetically differentiated from populations 3 and 4 (F_ST_ > 0.27; **Figure 2**). Similar analyses of populations 1 and 4, as well as 3 and 4 also identified high levels of genetic differentiation (F_ST_ = 0.26 and 0.25, respectively). Most of the improved sweet sorghum germplasm was distributed within population 2 (47%) and the admixed group (27%). Remarkably, population 1 consisted only of landraces.

Phylogenetic analysis indicated that the SWDP expands the genetic diversity present in the BAP (**Figure 2**). Multiple accessions from the SWDP clustered between the sweet sorghum accessions present in the BAP, suggesting that both panels could be combined to increase the statistical power to study sweet sorghum-related traits. Indeed, principal component analysis showed the genetic diversity within populations 2 and 3 is greater than the genetic diversity within sweet sorghum present in the BAP. The level of genetic differentiation between the high-biomass sorghums in the BAP and the SWDP was very low (F_ST_ = 0.02). Nevertheless, most of the SWDP germplasm forms a distinct cluster from the BAP in the phylogenetic analysis. Therefore, the SWDP contains additional genetic variation that could also be used to increase the statistical power of detection of under-represented alleles in the BAP relevant for the improvement of high-biomass sorghums.

The anthracnose resistance response was associated with SWDP population structure. When comparing the ratio of resistant to susceptible accessions at each field location where the SWDP was grown, we found evidence of selection against pathotypes from Georgia and Florida (**Table 1**). The overall frequency of resistant accessions in Georgia (0.30) was lower than that observed in population 1 (0.59) and 3 (0.54). Likewise, in Florida the overall frequency of resistant accessions (0.36) was lower than that observed in population 1 (0.63) and 3 (0.66). Remarkably, an excess of susceptible accessions in population 2 was observed in Florida (0.88) and Georgia (0.90). The ratio observed in Puerto Rico, Texas and across the four locations showed no evidence of selection for anthracnose resistance. The twenty-six accessions that were consistently resistant across locations represent the four populations present in the SWDP. Likewise, the photoperiod-sensitivity of accessions was associated with the SWDP population structure and anthracnose resistance response (**Supplementary Table 1**). We observed that 98 accessions did not reach flowering stage in Georgia, 46 of which (47%) were resistant to anthracnose. In contrast, only 19 accessions (16%) of the 123 that flowered were resistant to anthracnose. We observed selection for photoperiod-sensitive germplasm in populations 1, 3 and 4, and selection against photoperiod-sensitivity in population 2. The admixture group showed no evidence for selection for flowering time.

### Genome-wide association analysis for anthracnose resistance response in the SWDP

The photoperiod-sensitivity of the SWDP was first analyzed to validate the accuracy of the SNP data and their potential use in GWAS. The analysis identified four genomic regions in chromosomes 4, 6 and 7 associated with flowering time variation (**Supplementary Table S3**). The positive effects of the loci S4_53762801 (*p*-value = 2.67 ×10^-8^) and S6_39401690 (*p*-value = 3.44 ×10^-10^)] indicate that both are associated with photoperiod response, while the negative effects of the loci S4_66999721 (*p*-value = 3.97 ×10^-9^) and S7_16432373 (*p*-value = 6.39 ×10^-9^)] are likely to be related to the flowering time variation among photoperiod-insensitive accessions. Indeed, the two loci associated with photoperiod-sensitive responses colocalized with the flowering genes *Ma1* (Chr.6; Sobic.006G057866) and *Hd1* (Chr.4; Sobic.004G216700) (Murphy et al. (2011); **Supplementary Figure S2**), which explained 21.6% of the phenotypic variance. The analysis of Q-Q plots indicated that the BLINK model including the first three ancestry coefficients from STRUCTURE (K=4) provided an adequate control of the population structure and family relatedness.

**Figure 3:**
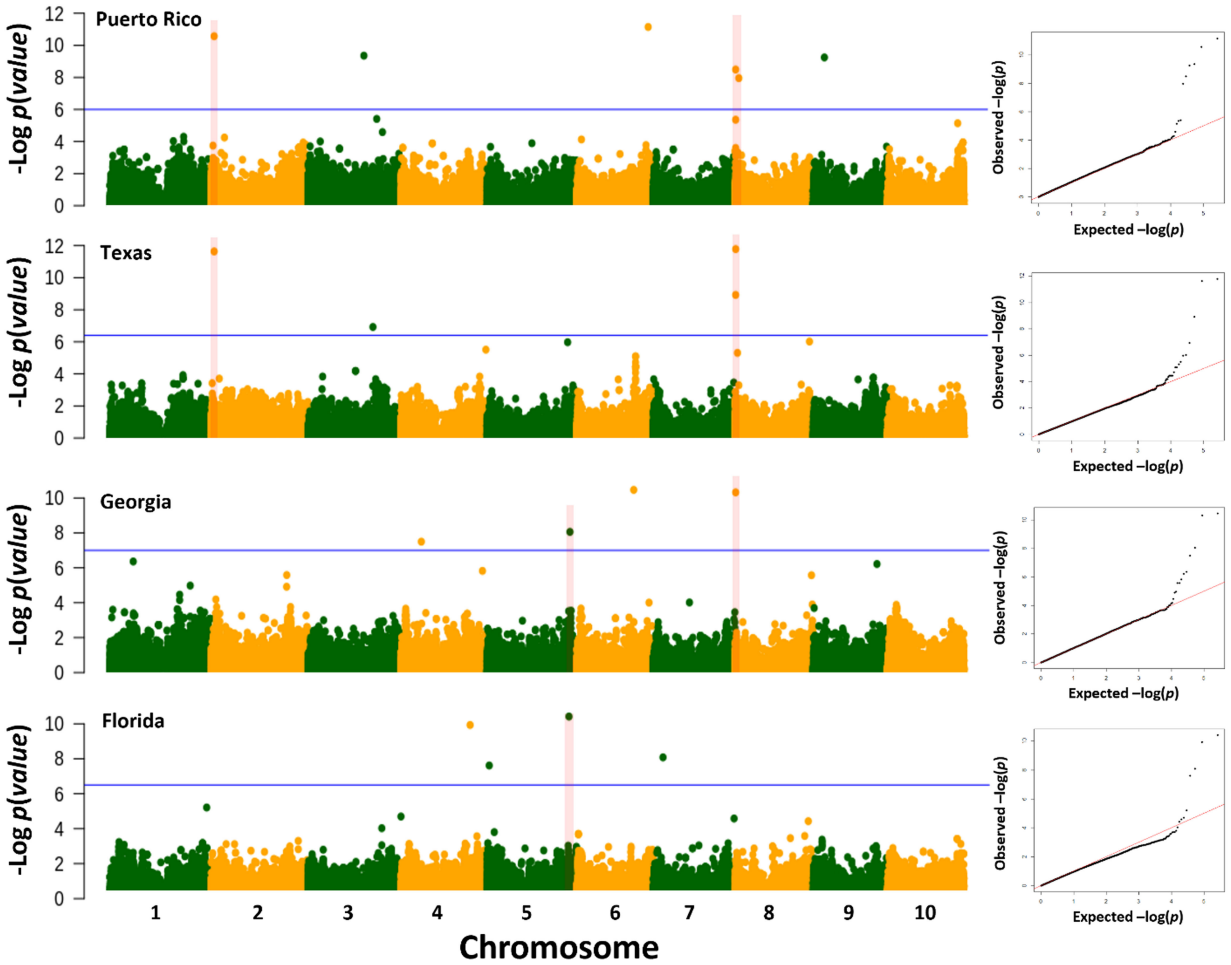
Genome-wide association analysis of anthracnose resistance in the NPGS sweet sorghum diversity panel. Manhattan plots for the BLINK association model using anthracnose resistance response observed in Florida, Georgia, Texas, and Puerto Rico in 2017 and 2018. The blue horizontal line refers to the false discovery rate threshold (Benjamini and Hochberg, 1995) and the red vertical line to common regions across locations. To the right of each Manhattan plot is the corresponding Q-Q plot as evidence that the inclusion of the first three ancestry coefficients adequately controlled for the population structure and family relatedness.

The genome-wide association scan for anthracnose resistance detected 16 loci across locations (**Figure 3**; **Table 3**). The largest number of associations were identified in Puerto Rico, with six resistance loci on chromosomes 2 (S2_2625340), 3 (S3_4470322), 6 (S6_57256182), 8 (S8_575263 and S8_3139785) and 9 (S9_8900776), which together explained 30.5% of the phenotypic variation. We detected four loci effective against pathotypes from Texas on chromosomes 2 (S2_262340), 3 (S3_51933644) and 8 (S8_575263, S8_616316), which together explained 23.7% of the phenotypic variation. The loci on chromosomes 2 (S2_2625340) and 8 (S8_575263) were identified in Puerto Rico and Texas, and thus provide protection against pathotypes from both locations. In Georgia, the genome-wide association scan identified four loci on chromosomes 4 (S4_16285663), 5 (S5_66523668), 6 (S6_45707450) and 8 (S8_585667), which together explained 39.0% of the phenotypic variation. The locus on chromosome 8 is 10.40 kb upstream of the locus S8_57263 identified in Texas and Puerto Rico. The lowest phenotypic variation explained (11.6%) was observed against the pathotypes of Florida. The GWAS detected four loci on chromosomes 4 (S4_55304023), 5 (S5_1971654 and S5_65730857) and 7 (S7_7905713). The loci S5_65730857 and S5_66523668 detected in Florida and Georgia, respectively, are separated by only 0.8 Mb.

**Table 3:** Genomic regions associated with anthracnose resistance response in the NPGS sweet sorghum diversity panel evaluated in Florida, Georgia, Texas, and Puerto Rico in 2017 and 2018.

	SNP			LD block^1^		
Location	Chr.	Position	*P*-value	Start	End	Interval (kb)
Puerto Rico	2	2625340	2.70 × 10^-11^	2618959	2625340	6.38
	3	44703822	4.42 × 10^-10^	44703822	44703831	0.01
	6	57256182	7.16 × 10^-12^	45670832	45707450	36.62
	8	575263	3.24 × 10^-9^	575183	587697	12.52
	8	3139785	1.11 × 10^-8^	3139785	3140238	0.45
	9	8900776	5.68 × 10^-10^	8899669	8924373	24.71
Texas	2	2625340	2.31 × 10^-12^	2618959	2625340	6.38
	3	51933644	1.19 × 10^-7^	51933644	51933668	0.03
	8	575263	1.19 × 10^-9^	575183	587697	12.52
	8	616316	1.65 × 10^-12^	611139	616316	5.18
Florida	4	55304023	1.17 × 10^-10^	55263175	55308581	45.41
	5	1971654	2.43 × 10^-8^	1971438	1971654	0.22
	5	65730857	3.84 × 10^-11^	65730834	65731631	0.80
	7	7905713	8.32 × 10^-9^	7905694	7905713	0.02
Georgia	4	16285663	3.19 × 10^-8^	16241104	16300392	50.29
	5	66523668	8.79 × 10^-9^	66496586	66523702	27.12
	6	45707450	3.45 × 10^-11^	45670832	45707450	36.62
	8	585667	4.78 × 10^-11^	575183	587697	12.52

^1^Linkage disequilibrium block determined with PLINK.

Genome-wide association analysis performed in GAPIT using the BLINK model.

The size of the LD blocks of these 16 loci varied across the genome. For instance, the five loci on chromosomes 4, 5 and 6 contained LD blocks larger than 25 kb. However, the LD block for the loci S8_575263 and S8_585667 detected in Georgia, Texas and Puerto Rico were smaller than 12.5 kb. We did not detect a correlation between the size of the LD blocks and the effect of these loci.

### Candidate genes

**Figure 4:**
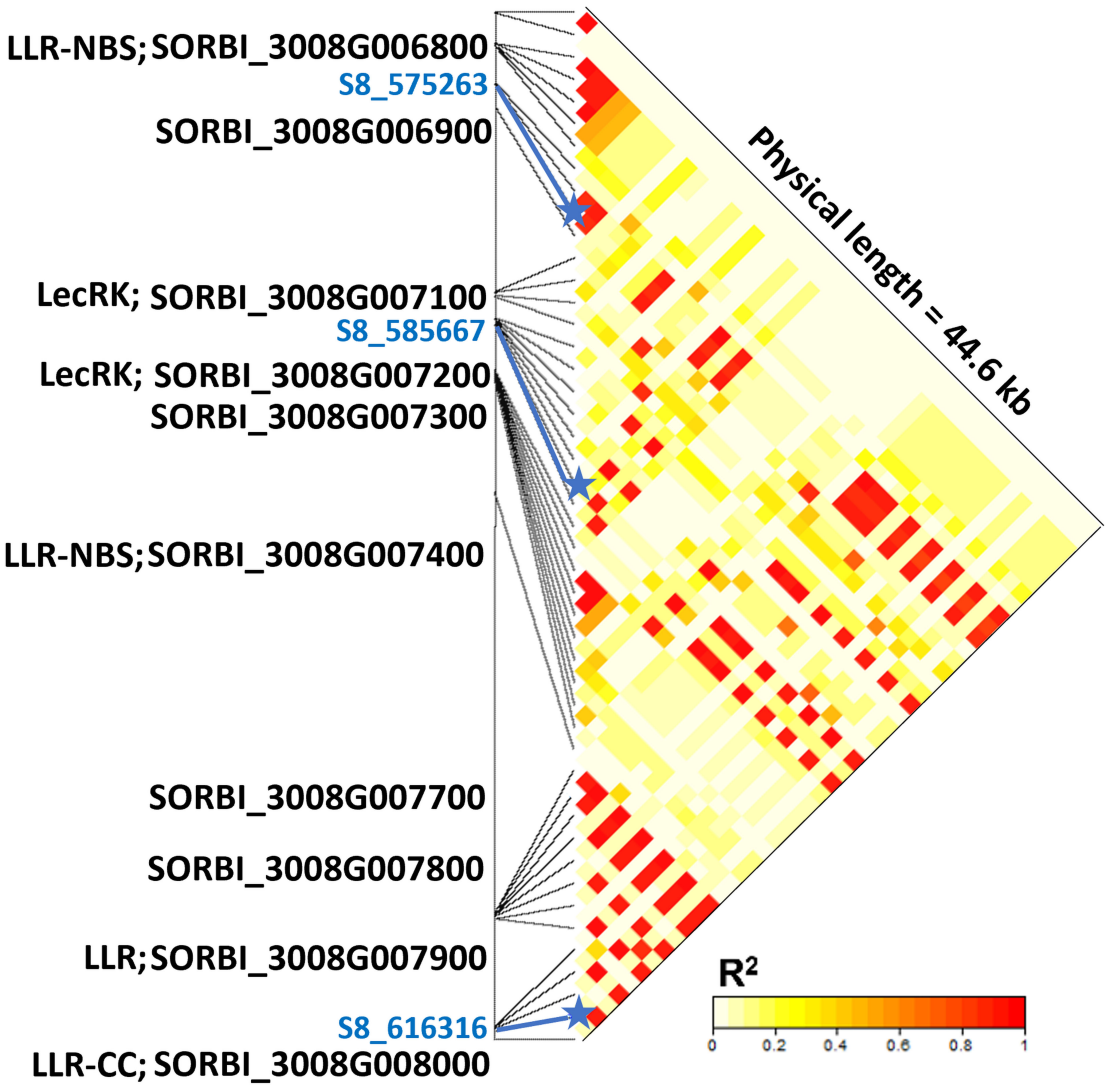
Linkage disequilibrium and candidate genes within a 44.6 kb genomic region of chromosome 8 associated with anthracnose resistant response observed in a sweet sorghum diversity panel evaluated in Texas, Georgia, and Puerto Rico. The SNP S8_575263 is associated with resistance responses observed in Texas and Puerto Rico, while SNPs S8_585667 and S8_616316 are associated with resistance responses observed in Georgia and Texas, respectively. Protein domains encoded by candidate resistance genes are indicated with the following abbreviations: LRR, leucine rich repeats; NBS, nucleotide binding site; CC, coiled coil; and LecRK, lectin receptor kinase.

The resistance response against pathotypes from Texas and Puerto Rico mapped to the distal end of the short arm of chromosome 8. This region contained multiple *R* genes (**Figure 4**). For instance, the LD block containing the SNPs S8_575263 and S8_585667 is 1.6 kb downstream of gene *Sobic.008G006800*, which encodes leucine-rich repeats (LRR) and protein kinase receptor domains. Similarly, the LD block containing the SNP S8_616316 harbors the genes *Sobic.008G007900* and *Sobic.008G008000*, both encoding LRR domains. In fact, six of the ten genes predicted in this 41 kb region have features related to disease resistance.

We identified two LD blocks on chromosome 5 associated with resistance against pathotypes from Florida and Georgia that harbor disease resistance genes. The SNP S5_65730857 and its 800 bp LD block on chromosome 5 are located in gene *Sobic.005G175500*, which is annotated to encode a protein tyrosine kinase domain implicated in antifungal activity. Likewise, the 27.12 kb block that contains SNP S5_66523668 harbors only one annotated gene (*Sobic.005G182500.2*) that encodes protein kinase and Jacalin-like lectin domains, both often associated with disease resistance responses.

## Discussion

The NPGS sweet sorghum collection is a publicly available germplasm collection that has not been genetically and phenotypically characterized for biofuel-related traits (Cuevas et al., 2015). The identification of the most valuable sweet sorghum germplasm will expedite the development of new sweet sorghum cultivars and hybrids by avoiding time-consuming introgression breeding approaches with non-sweet sorghums (i.e. grain, forage and high-biomass germplasm). Today, the BAP is the most important germplasm and genomic resource to advance the breeding of bioenergy sorghums (Brenton et al., 2016). Most of its accessions are high-biomass sorghums, as opposed to sweet sorghums, and have resistance to major foliar diseases. This selection bias in the BAP reduces the statistical power in GWAS to detect alleles associated with resistance against foliar diseases, including anthracnose. We established a publicly available sweet sorghum diversity panel as a novel resource for the mining of sorghum disease resistance alleles and other biofuel-related traits, which also expands the genetic diversity of sweet sorghum germplasm in the BAP. We observed that the phenotypic and genetic diversity in this novel diversity panel is also suitable for the genomic dissection of complex traits. For instance, the GWAS for flowering time detected loci not previously identified in temperate adapted grain sorghum, suggesting the panel could also be of interest for the identification of other important genes.

The actual number of sweet sorghum accessions in the NPGS sorghum collection is unknown and its identification requires extensive phenotyping analysis. The level of genetic differentiation between sweet, high-biomass and grain sorghum germplasm is not well understood and is considered polyphyletic (Ritter et al., 2007; Brenton et al., 2016). Resequencing analysis of a few sweet and grain lines has revealed genetic variation in many genes involved in sugar production (Zheng et al., 2011). Likewise, the comparison of the reference genomes of BTx623 (grain type) and Rio (sweet sorghum type) identified high levels of structural similarity but also a high number of mutations in transcriptionally active genes (Cooper et al., 2019). The *Dry* locus, which controls pithy versus juicy stems and is a major factor differentiating sweet sorghum from other types, was recently mapped to a NAC transcription factor gene on chromosome 6 (Zhang et al., 2018). The further identification of genetic profiles in sweet sorghum germplasm could be used for screening the NPGS sorghum collection to identify additional sweet sorghum accessions to expand the SWDP.

Previous genetic analysis of NPGS sweet sorghum germplasm based on a limited number of SNP or simple sequence repeat (SSR) markers revealed limited genetic diversity amongst most of the U.S. improved germplasm (Murray et al., 2009). Nevertheless, our whole-genome characterization showed that the average genetic distance between accessions in the SWDP (0.64) was similar to that observed in the BAP (Brenton et al., 2016) and lower than in Ethiopian and Sudanese NPGS collections [0.67 and 0.74, respectively; (Cuevas et al., 2017; Cuevas and Prom, 2020)], indicating that the SWDP has broad genetic diversity. To obtain insight into the genetic diversity present within the 99 improved sweet sorghum accessions of the SWDP we analyzed them separately. The pairwise genetic distance indicated a close genetic relationship amongst these accessions. In fact, the 47 improved accessions present in population 2 were highly related genetically, with an average genetic distance of 0.78. The integration of additional accessions that will broaden the overall genetic diversity is, therefore, necessary for the success of future sweet sorghum breeding programs based on the SWDP. In this regard, the population structure and phylogenetic analysis presented herein outline genetic distances among accessions that may be exploited through investigations of heterosis. Certainly, the 26 anthracnose resistant accessions are highly genetically diverse and can be used in recurrent selection breeding programs to uncover additional genetic variation.

Most of the U.S. sweet sorghum germplasm was developed to be cultivated in the Southeast, Mid-South and lower Midwest, where anthracnose is an endemic disease. Resistance to foliar and stalk diseases were considered important traits due to their large negative effects on the yield and quality of sweet sorghum syrup. In fact, we observed that the majority of the advanced sweet sorghum germplasm was resistant to anthracnose in Texas and Puerto Rico (52% and 61%, respectively), while in Florida and Georgia only 21% of the advanced germplasm was resistant. The existence of multiple *C. sublineolum* pathotypes (Prom et al., 2012) across locations is the most plausible explanation for the observed variation in the resistance response across locations. Moreover, the GWAS confirmed that resistant alleles effective against pathotypes from Puerto Rico and Texas are present at higher frequency in the SWDP than those against pathotypes from Florida. The narrow genetic diversity present among the resistant accessions within the advanced germplasm suggests most of them have similar resistance mechanisms (i.e. many loci are identical-by-descent). Possibly, the resistance loci at the distal ends of the short arms of chromosomes 2 and 8 identified in Texas and Puerto Rico were inherited from one or two accessions used extensively in breeding programs. This limited number of resistance sources amongst advanced sweet sorghum germplasm exposes its vulnerability to the rapid evolution of the pathogen. Landraces resistant to anthracnose in the SWDP, particularly from population 1, are likely to harbor novel resistance sources that could be incorporated into sweet sorghum breeding programs to increase the durability of the resistance.

Comparative genome mapping analysis revealed that two of the genomic regions identified in our study have been previously associated with anthracnose resistance in both biparental and GWAS studies. Two loci on chromosome 5 (SNPs S5_65730557 and S5_66523668) associated with resistance against pathotypes from Florida and Georgia, respectively, are located within a 1.83 Mb region (64.69 -66.52 Mb) that has been associated with anthracnose resistance in two grain sorghum lines (SC414-12E and SC112-14), the SAP and the NPGS Sudanese core collection (Patil et al., 2017; Cuevas et al., 2018; Cuevas and Prom, 2020). The LD blocks of both loci are 76.1 kb downstream and 4.8 kb upstream of SNP S5_66491767, which was associated with anthracnose resistance in the SAP (Cuevas et al., 2018). We also determined that the 45.4 kb resistance locus on chromosome 4 identified in Florida is located within a large QTL region identified in the lines SC155-14E and SC414-12E (Patil et al., 2017). Certainly, the SWDP can be used for both the identification of novel anthracnose resistance loci and the genomic dissection of other resistance loci identified in grain sorghum. Its further integration with other diversity panels (e.g. BAP, SAP) could led to the discovery of novel common resistance loci.

Candidate gene analysis for the 16 anthracnose resistance loci identified via GWAS indicated that both membrane-associated receptors and signaling cascades are implicated in the observed resistance responses. The large genetic diversity of the pathogen (Prom et al., 2012) and the presence of different molecular defense mechanisms suggest that the introgression of multiple resistance loci will be necessary to ensure a durable resistance response that is effective across a wide geographic area. To obtain insight in the role of the 16 loci identified in this study, we compared genetic profiles in the 26 resistant (**Table 4**) and 57 susceptible accessions across locations. We first tallied the presence of resistance alleles at each locus and observed that it ranged from 8 to 15 and from 3 to 12 in resistant and susceptible accessions, respectively. The average was slightly higher in the resistant group (11 loci) than in the susceptible group (9 loci). We subsequently compared the frequency of resistance alleles for each locus in both groups. This analysis showed that the frequency of resistance alleles at 12 loci was higher in the resistant group than in the susceptible group. Remarkably, nine of these loci showed allele frequency differences greater than 0.20. Therefore, the selection of resistant accessions for breeding programs should be based on population structure and the genetic profile for these 16 loci. In this regard, the accessions PI524891 (Population 1), PI648090 (Population 2) and PI648099 (admixed) could be classified as the most suitable germplasm for the introgression of anthracnose resistance alleles in sweet sorghum breeding programs.

**Table 4:** Genetic profiles of sixteen anthracnose resistance loci in 26 accessions of the NPGS sweet sorghum diversity panel (SWDP) that showed a resistant response in Texas, Georgia, Florida and Puerto Rico for two consecutive years.

		Anthracnose resistance loci in SWDP																
Population^1^	ID	S2_2625340	S3_44703822	S3_51933644	S4_16285663	S4_55304023	S5_1971654	S5_65730857	S5_66523668	S6_45707450	S6_57256182	S7_7905713	S8_3139785	S8_575263	S8_585667	S8_616316	S9_8900776	
Pop. 1	PI_155608	+/+	-/-	+/+	+/+	+/+	-/-	+/+	-/-	-/-	+/+	+/+	+/+	-/-	-/-	+/+	-/-	
Pop. 1	PI_155631	+/+	n.a.	+/+	+/+	+/+	-/-	+/+	-/-	-/-	+/+	+/-	+/+	-/-	-/-	+/+	+/+	
Pop. 1	PI_155796	+/+	-/-	+/+	+/+	+/+	-/-	+/+	+/+	-/-	+/+	+/-	+/+	+/+	-/-	+/+	+/+	
Pop. 1	PI_156238	+/+	-/-	+/+	+/+	+/+	-/-	+/+	+/-	-/-	+/+	-/-	+/+	+/+	+/+	+/+	-/-	
Pop. 1	PI_156356	+/+	-/-	+/+	+/+	+/+	-/-	+/+	-/-	-/-	-/-	+/-	+/+	-/-	-/-	+/+	-/-	
Pop. 1	PI_156360	+/+	-/-	+/+	+/+	+/+	-/-	-/-	-/-	-/-	-/-	+/-	+/+	-/-	-/-	+/+	+/+	
Pop. 1	PI_524891	+/+	+/+	+/-	+/+	+/+	+/-	+/+	+/+	-/-	+/+	+/-	+/+	+/+	+/+	+/+	+/-	
Pop. 2	PI_641914	+/+	+/+	+/+	+/+	+/+	-/-	-/-	+/+	-/-	+/+	-/-	+/+	+/+	+/+	+/+	-/-	
Pop. 2	PI_648090	+/+	+/-	+/+	+/+	+/+	+/+	+/+	+/-	-/-	+/+	+/-	+/+	+/+	+/+	+/+	-/-	
Pop. 2	PI_651363	+/+	n.a.	+/+	+/+	+/+	-/-	-/-	+/+	-/-	-/-	-/-	+/+	+/+	+/+	+/+	+/+	
Pop. 2	PI_651373	+/+	n.a.	+/+	+/+	+/+	-/-	-/-	+/+	-/-	+/+	-/-	+/+	+/+	+/+	+/+	-/-	
Pop. 3	PI_152969	+/+	+/-	+/+	+/+	+/+	+/+	+/+	-/-	-/-	+/-	-/-	-/-	-/-	-/-	+/+	+/+	
Pop. 3	PI_641909	+/+	+/+	-/-	+/+	+/+	+/+	+/+	-/-	-/-	-/-	+/+	+/+	-/-	-/-	+/+	+/+	
Pop. 3	PI_651333	+/+	+/-	+/+	+/+	+/+	-/-	+/+	-/-	-/-	+/+	+/-	+/+	-/-	-/-	+/+	+/+	
Pop. 3	PI_651409	+/+	+/-	+/+	+/+	+/+	+/+	+/+	-/-	-/-	-/-	-/-	+/+	+/+	+/+	+/+	-/-	
Pop. 4	PI_273971	+/+	-/-	+/+	+/+	+/+	-/-	n.a.	+/-	-/-	+/+	+/-	+/+	-/-	-/-	+/+	+/+	
Pop. 4	PI_330829	+/+	n.a.	+/+	+/+	+/+	-/-	+/+	-/-	-/-	+/+	+/-	+/+	-/-	-/-	+/+	+/+	
Pop. 4	PI_648132	+/+	n.a.	+/+	+/+	+/+	+/+	n.a.	-/-	-/-	+/+	+/-	+/+	-/-	-/-	+/+	+/+	
Admixed	PI_155230	+/+	-/-	+/+	+/+	+/+	+/+	+/+	-/-	-/-	-/-	+/-	+/+	-/-	-/-	+/+	-/-	
Admixed	PI_196072	+/+	+/+	+/+	+/+	+/+	-/-	-/-	+/-	-/-	+/+	+/-	+/+	+/+	+/+	+/+	-/-	
Admixed	PI_648086	+/+	n.a.	+/+	+/+	+/+	-/-	-/-	+/+	-/-	+/+	-/-	+/+	-/-	-/-	-/-	+/+	
Admixed	PI_648099	+/+	+/+	+/+	+/+	-/-	+/+	+/+	-/-	-/-	+/+	+/-	+/+	+/+	+/+	+/+	+/+	
Admixed	PI_651369	+/+	n.a.	+/+	+/+	+/+	-/-	-/-	+/-	-/-	-/-	+/-	+/+	+/+	+/+	+/+	-/-	
Admixed	PI_651370	+/+	+/+	+/+	+/+	+/+	-/-	-/-	+/-	-/-	-/-	+/-	+/+	+/+	+/+	+/+	-/-	
Admixed	PI_651393	+/+	+/+	+/+	+/+	+/+	-/-	+/+	-/-	-/-	+/+	+/-	+/+	-/-	-/-	+/+	+/+	
Admixed	PI_653616	+/+	n.a.	+/+	+/+	+/+	-/-	-/-	+/+	-/-	+/+	-/-	+/+	+/+	+/+	+/+	+/+	

^1^Population structure analysis of the SWDP using 2,345 unlinked genome-wide SNPs.

## Data availability statement

The original contributions presented in the study are included in the article/**Supplementary Files**, further inquiries can be directed to the corresponding author/s.

## Author contributions

HC: Conceptualization; Formal analysis; Funding acquisition; Investigation; Methodology; Writing-original draft, reviewing and editing. JK: Formal analysis; Funding acquisition; Investigation; Methodology; Writing-reviewing. LP: Formal analysis; Funding acquisition; Investigation; Methodology; Writing-reviewing. LS: Investigation, Writing-reviewing. WV: Formal analysis; Funding acquisition; Investigation; Methodology; Writing-reviewing. All authors contributed to the article and approved the submitted version.
