# Supplementary material for: Genetic diversity, population structure and anthracnose resistance response in a novel sweet sorghum diversity panel
Source: Front Plant Sci. 2023 Oct 20;14:1249555. doi: 10.3389/fpls.2023.1249555 (PMC10623324; doi:10.3389/fpls.2023.1249555)
Supplement: Supplementary file 1 [file DataSheet_1.docx]

**
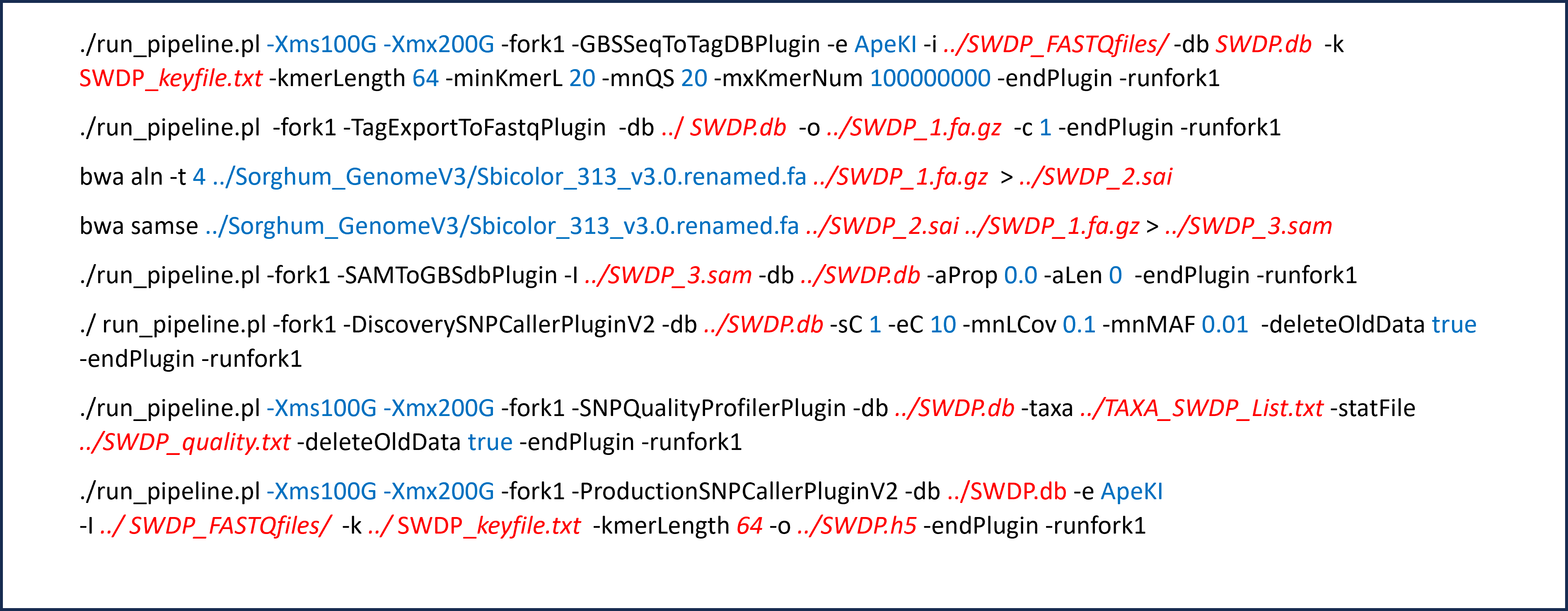
Supplementary Figure S1**. Tassel scripts for SNP call for the GBS analysis of the 272 accessions present in the NPGS sweet sorghum diversity panel.

**Supplementary Table S3** Genomic regions associated with flowering time variation in NPGS sweet sorghum diversity panel evaluated in Georgia in 2017 and 2018. Genome-wide association analysis performed in GAPIT using the BLINK model.

| **Chromosome** | **Position** | ***P*-value** | **MAF^1^** | **H&B *P*-value^2^** | **SNP Effects** |
| --- | --- | --- | --- | --- | --- |
| 6 | 39401690 | 3.44×10^-10^ | 0.46 | 4.6×10^-5^ | 48.32 |
| 4 | 66999721 | 3.97×10^-9^ | 0.46 | 2.6×10^-4^ | -28.20 |
| 7 | 16432373 | 6.39×10^-9^ | 0.08 | 2.8×10^-4^ | -52.92 |
| 4 | 53762801 | 2.67×10^-8^ | 0.16 | 8.8×10^-4^ | 29.09 |

^1^ Minor allele frequency

^2^ P-value for the Holm-Bonferroni method


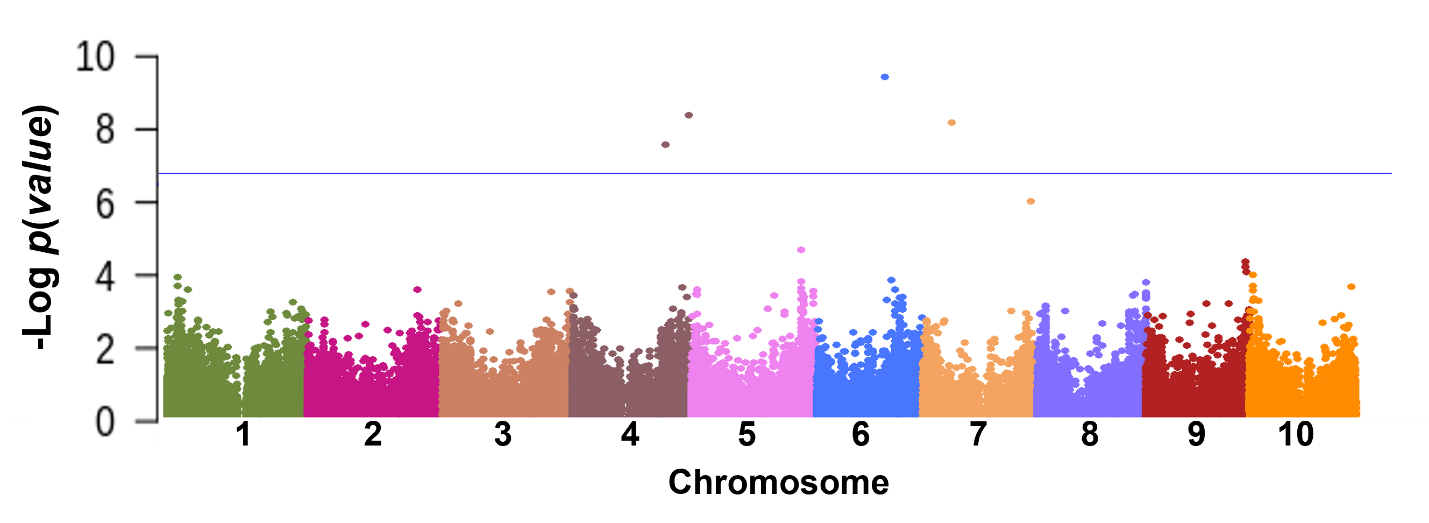
**Supplementary Figure S2**. Genome-wide association analysis of flowering time in the NPGS sweet sorghum diversity panel evaluated in Georgia in 2017 and 2018.The blue horizontal line refers to the false discovery rate threshold.
